# The effect of promotional health message framing on the perceived benefit of mammography: evidence from estimation of willingness to pay

**DOI:** 10.1186/s41043-025-00970-8

**Published:** 2025-06-21

**Authors:** Bahman Ahadinezhad, Gita Rashvand, Simin Karimi, Ahad Alizadeh, Mahammad Amerzadeh, Omid Khosravizadeh

**Affiliations:** 1 Social Determinants of Health Research Center, Research Institute for Prevention of Non-Communicable Diseases, Qazvin University of Medical Sciences, Qazvin, Iran; 2 Student Research Committee, School of Public Health, Qazvin University of Medical Sciences, Qazvin, Iran; 3 Non-Communicable Diseases Research Center, Research Institute for Prevention of Non-Communicable Diseases, Qazvin University of Medical Sciences, Qazvin, Iran

**Keywords:** Willingness to pay, Framing effect, Mammography

## Abstract

**Background:**

Evidence from behavioral economics has shown that framing health information can impact the demand for screening. We examined the effect of promotional message framing on mammography demand by estimating willingness to pay (WTP).

**Methods:**

This experimental study was conducted in 2024 over a period of 3 months. The interesting outcome was a WTP for mammography. 354 women were randomly selected and randomly assigned between the two study arms. The intervention involved the presentation of health information in two frames of gain and loss. The mammography demand has been estimated using robust standard error Logistic regression. Demand rate of mammography has been compared between two types of information framing using the chi-square test. Finally, the monetary value of willingness to pay (WTP) for mammography was estimated using the methodology developed by López-Feldman. All analyses were done using STATA 17.

**Results:**

The price and income elasticity of mammography demand were estimated as − 0.19 and 0.24, respectively (*P* < 0.01). The higher demand rate in the loss frame compared to the gain frame (38.7% vs. 25.1%) was statistically significant, and its effect size was estimated to be 0.282 (*p* < 0.01). The value of WTP in the loss frame (10.68 US$) was estimated to be more significant than in the gain frame (4.74 US$) (*p* < 0.01).

**Conclusion:**

This study suggests that health educators consider the message’s persuasiveness with the type of health action before designing health messages. Moreover, health practitioners should use health messages with a loss frame to increase the demand for screening services such as mammography.

**Supplementary Information:**

The online version contains supplementary material available at 10.1186/s41043-025-00970-8.

## Background

During the last four decades, the incidence of breast cancer has increased alarmingly [1]. Approximately one in four women may develop breast cancer in their lifetime, and one in eight may die from the cancer [2]. In 2020, approximately 2.3 million new cases of breast cancer were recorded worldwide, and approximately 685,000 women died from this disease [3]. In Iran, the incidence of breast cancer in women is increasing rapidly and constitutes 28.1% of women’s malignancies [4]. In 2020, 16,967 new cases of breast cancer and 4810 deaths were reported in Iran [5]. In 2019, breast cancer resulted in more than 187 million DALYs worldwide [6].

In Iran, a standardized, nationwide breast cancer screening program utilizing mammography has not yet been implemented. Women eligible for screening generally obtain mammography services through private healthcare providers or by referral from comprehensive health centers. Current data suggests that the costs associated with these screenings are not fully subsidized by the government, with a portion of the expenses being covered by health insurance providers.

Medical imaging techniques such as mammography are widely used for breast cancer screening [7, 8]. The U.S. Preventive Services Task Force recommends biennial screening mammography for women aged 40–74 years [9]. Mammography can be a valuable screening for breast cancer diagnosis, and it can help prevent tumor spread and improve treatment outcomes through timely diagnosis [10]. Reducing the death rate due to breast cancer as a result of mammography is cost-effective in improving women’s health [11]. The European guidelines also recommend that at least 70–75% of women participate in regular mammography screening [12].

Based on decision-making models, people perform a subjective cost–benefit analysis regarding the demand for healthcare services such as screening [13]. Therefore, the perceived benefit can affect the demand for mammography [14–16]. Bakhtiari [17] found that not having a good understanding of the benefits of mammography was one critical obstacle to performing mammography. Evidence from behavioral economics has shown that the messages framing and health information can impact the acceptance and performance of screening [18].

The framing of health messages includes the choice of words, the way information is presented, and the types of emotions and images conveyed to the audience. Research has shown that health messages, if designed properly, can create greater motivation for people to adopt positive health behaviors. In this regard, questions such as which type of framing can have the greatest impact on women’s decision to have a mammogram are important. Message framing is an influential communication approach to influence people’s attitudes and behaviors [19]. For example, loss-framed messages are more effective in reducing risky behaviors, while gain-framed messages are more effective in increasing safe behaviors [19]. A meta-analysis study by Ahdinezhad et al. [20] revealed that message framing can affect improving colorectal cancer screening rates. A systematic review and meta-analysis [21] found that loss-based messages have achieved initial success in persuading people to take up cancer screening behaviors.

Prospect theory [22] suggests that losses are more prominent than gains in human judgment. Several studies have investigated the effect of this framing on health behaviors. In a meta-analysis, Gallagher & Updegraff [23] found that gain-framed messages encouraged prevention behaviors such as skin cancer prevention, smoking cessation, and physical activity more than loss-framed messages. Maltz et al. [24] concluded that there was no significant difference in the rate of medical checkup uptake between the gain and loss frames. The evidence obtained from studies is mixed, and the effectiveness of framing based on perspective theory in health is debated [23, 25, 26].

Studies in Iran have examined the effectiveness of promotional messages on screening behaviors [27–30], but based on our search, none examined the effects of message frame type. In this paper, we aim to contribute to covering this gap in literature and empirical evidence. The issue we address in this study is the answer to the question of which type of health message frame (loss or gain) has a greater impact on the likelihood of demanding the mammography and its perceived benefit? Therefore, this study aimed to estimate the willingness to pay (WTP) for mammography among women in Qazvin City and the effect of the type of health message frame on the demand for mammography.

## Methods

### Study design and settings

This quasi-experimental research was conducted cross-sectionally and through a population survey. It was completed in Qazvin City (the capital of the Qazvin province), 150 km from Tehran (the capital of Iran). Data collection was carried out in city parks, which were designated as the survey sites.

#### Study population and sampling

Our research population comprised Qazvin women aged 40 to over 70 years old. The U.S. Preventive Services Task Force recommends biennial screening mammography for women aged 40–74 years [9]. A total of 354 women were selected using multi-stage sampling, specifically employing a cluster-availability sampling method. In the first stage, the target population was clustered based on the city’s urban regions (1, 2, and 3), and the parks within each region were identified. (Appendix A2). In the second stage, two parks were randomly selected from each region. In the third stage, the proportion of the total female population over 40 years of age in each geographical region was determined. It was obtained from population data covered by the Comprehensive Health Center. In the fourth stage, the proportional sample size for each region was calculated based on the proportion of the female population over 40 years of age in each region. (By dividing the number of women over 40 in each area by the total population over 40 in the city). Finally, in the last stage, women from each region who were willing to participate were selected as the sample population.

We have estimated the sample size using the following formula with parameters α = 0.05, d = 0.1p, p = 0.539:

$$n = \frac{{\overline{z}_{1 - \alpha /2}^{2} \times p(1 - p)}}{{d^{2} }}$$

We obtained the rate of mammography uptake (p) from Farzaneh et al. [26].

The inclusion criteria were living in Qazvin City, being 40 to over 70 years old, being willing to participate, and having cognitive ability. Women under 40 years of age, living outside the city of Qazvin, with breast cancer, were not included in the study. Participants were recruited from 20 February to 15 March 2024.

#### Randomization

Participants were randomly allocated to one of two experimental conditions (loss frame or gain frame) through the use of a random number table.

### Data collection

#### Collection tools

The required data was collected using a questionnaire in the form of a face-to-face interview. The questionnaire consists of two parts: (1) an assessment of participants’ demographic and socio-economic information (see Appendix A) and (2) a willingness-to-pay (WTP) question (see Appendix C). The willingness to pay question is designed based on the Fleischman Foreit & Foreit (2004) guidelines [31]. In the willingness to pay question, each participant is offered an initial price and then asked whether or not she would demand a mammography at the offered price. To address potential biases arising from initial price anchoring effects, the bid price was randomly selected from a predefined vector of prices. This vector was developed based on a survey of three prevalent market prices for mammography. The selected prices, which represent out-of-pocket costs, were $1.17, $3.33, and $9.67. These amounts correspond to the insurance deductible, government tariff, and private tariff for mammography, respectively. The pricing data were obtained from the tariff table available at the time of data collection. A repeated vector of these three prices was generated using a random number generation process. Table 1 outlines the two study arms (loss frame and gain frame), categorized according to the initially offered price.

**Table 1:** Matrix of 6 modes of the questionnaire

Bid (US$)	Gain frame	Loss frame
1.17	59 questionnaires	59 questionnaires
3.33	59 questionnaires	59 questionnaires
9.67	59 questionnaires	59 questionnaires

At the time of data collection, each US dollar was equivalent to 600,000 Iranian Rial

#### Collection procedures

By referring to the survey locations (parks) and introducing themselves, the interviewers asked the women to answer the questionnaire. The interviewers emphasized that the participant (female) herself answer the questions. First, each participant was asked about information related to demographic and socioeconomic characteristics. The participant was then randomly presented with one of the health information texts (gain or loss frame) and was allowed 10 min to read and reflect on the information. In the next step, a price was randomly drawn from the price vector and presented to the individual, who was asked to indicate whether or not she would demand a mammogram at the proposed price. The above steps were repeated for the entire sample size. The collected data was organized in an Excel file.

#### Intervention

The intervention involved the presentation of health information in two distinct frames: benefit (gain frame) and loss (loss frame), delivered to participants in textual format. Initially, comprehensive information about mammography was collected, including details on cancer detection, severity at diagnosis, and survival rates. Drawing on the work of Sun et al. [32], Leewon et al. [33], Lipkus et al. [34], and Stoffel et al. [35], the research team developed two narrative frameworks: one emphasizing the benefits of undergoing mammography and the other underscoring the potential losses associated with not undergoing the procedure [32–35]. The promotional material was provided to participants exclusively in text format, structured using the two frames of loss and gain (see Appendix B).

#### Study variables

The study variables were two groups. (1) Covariates include age, literacy level, activity status, health insurance status, monthly income, perceived economic status,^1^ and perceived risk. (2) The primary outcomes were the demand for mammography. To measure it, participants are asked to indicate whether they would performed mammography at the proposed price. Yes or no answers were coded with the 1 and zero, respectively. Willingness to pay is the amount of money that people are willing to pay to own a good or service. Here is the average currency (Rial and Dollar) obtained from estimating the demand function using the method explained in the econometrics section.

We asked participants to state their risk of breast cancer between zero and 100% to measure the perceived risk. Perceived economic status was also measured through a question. In this way, we asked people to determine their economic status on a Likert scale (very poor to very rich).

#### Data quality assurance

To ensure data quality, we used these strategies: In designing the questionnaire, we vexed to use simple, understandable, and unambiguous questions. We calculated the sample size accurately and attempted to use an unbiased sampling method. We trained the interviewers before collecting the data. After collecting the data, we reviewed the data and eliminated any incorrect or illogical data. We used appropriate statistical methods to analyze the data to obtain valid and reliable results.

#### Data processing and analysis

We analyzed the data in several steps. First, we organized the collected data in an Excel file and entered it into the STATA 17 software. The data were analyzed using a histogram to examine the experimental distribution. Since there were no missing data, it was not necessary to assess the missing data structure. In the second step, we described participants’ demographic and socioeconomic characteristics based on statistical indicators. In the third step, we reported the rate of mammography demand according to the offered prices. In the fourth step, we estimated the mammography demand price^2^ and income elasticities^3^ using robust standard error Logistic regression and extracted the demand curve. In the fifth step, we compared the mammography demand rate between two types of information framing by running the chi-square test. Finally, the monetary value of willingness to pay (WTP) for mammography was estimated using the methodology developed by López-Feldman [35]. The estimation was conducted through the application of three distinct econometric models.

#### Econometric model

To estimate participants’ willingness to pay, we employed the López-Feldman [35] method. This approach involved two key steps. First, the basic demand function for mammography was estimated using a probit regression model. Second, the average values of the explanatory variables, specifically income and price, were calculated, and a scalar was constructed for each. These steps formed the basis for deriving the WTP estimates. Then, through the formula WTP:$EWTP{|}\widetilde{Z, }\beta = \tilde{Z} \left[ { - \frac{{\hat{\alpha }}}{{\hat{\delta }}}} \right]$ the willingness to pay was estimated at a 95% confidence interval. The following model illustrates the mammography demand function based on binary probit regression. Let I denote the ith participant, and Y_i_ represent the mammography demand of the ith participant at the offered price and her income. Y_i_ is a binary variable that assumes values of 0 or 1. The variable X_i_ indicates the information vector comprising bid prices and participants’ income. β_0_ signifies the intercept of the demand function, while β represents the vector of coefficients reflecting the effects of bid price and income on demand. Additionally, ε_i_ denotes the error term, which is assumed to follow a normal distribution.

$$Y_{i} = \beta_{0} + \mathop \beta \limits^{\prime } X_{i} + \varepsilon_{i}$$

WTP can be modelled as the following:

$$WTP_{i} \left( {z_{i} ,u_{i} } \right) = z_{i} \beta + u_{i}$$

where z_i_ is a vector of explanatory variables, β is a vector of parameters and u_i_ is an error term. By running probit command in Stata we obtained estimates of β/σ and − 1/σ. σ represents the standard deviation of WTP.

Through $\hat{\alpha } = \frac{{\hat{\beta }}}{{\hat{\sigma }}} ,\;\hat{\delta } = \frac{1}{{\hat{\sigma }}}$, and using the following formula, we estimated the WTP:$EWTP{|}\widetilde{Z, }\beta = \tilde{Z} \left[ { - \frac{{\hat{\alpha }}}{{\hat{\delta }}}} \right]$.

### Ethical statement

Written informed consent was obtained from all samples. This research was carried out with the permission and certificate (IR.QUMS.REC.1402.371) of the Research Ethics Committee of Qazvin University of Medical Sciences (Appendix D).

## Results

### Socio-demographic and economic characteristics of the respondents

Table 2 describes participants’ demographic and socioeconomic characteristics. The average age of the participants was about 54 years, and most (42.4%) were between 40 and 50 years old. The ages of participants ranged from a minimum of 40 years to a maximum of 73 years. Most participants (36.7%) had a high school education. 74.3% were jobless. 94.4% had health insurance. 49.72% evaluated their economic status as middle. The average perceived risk was reported as 5.78% ± 18.1. Participants reported they earn an average of 211.7 ± 198.3 US$ per month.

**Table 2:** Socio-economic characteristics of the participants

Characteristic	Subgroup	Frequency	Percentage
Age	40–50	150	42.4%
	50–60	111	31.4%
	60–70	89	25.1%
	> 70	4	1.1%
Literacy level	Illiterate	21	5.9%
	High school	130	36.7%
	Diploma	77	21.8%
	Associate Degree	35	9.9%
	Bachelor’s degree	66	18.6%
	Master’s degree	21	5.9%
	Doctorate	4	1.1%
Employment status	Jobless	263	74.3%
	Employed	91	25.7%
Perceived economic status	Very poor	43	12.15%
	Poor	88	24.86%
	Middle	176	49.72%
	Rich	44	12.43%
	Very rich	3	0.85%

Characteristic	Mean	SD
Age (Year)	53.9	8.8
Perceived risk (%)	5.78	18.1
Monthly total income (US$)	211.7	198.3
Initial bid (US$)	4.76	3.63

At the time of data collection, each US dollar was equivalent to 600,000 Iranian Rial

The results comparing participant characteristics between the two study arms, gain and loss, are detailed in Table E1 of Appendix E. As shown in the table, no statistically significant differences were observed in participant characteristics between the two groups. This finding supports the effectiveness of the randomization process in ensuring comparability between the study arms.

### Results of Mammography demand estimation

**Fig. 1:**
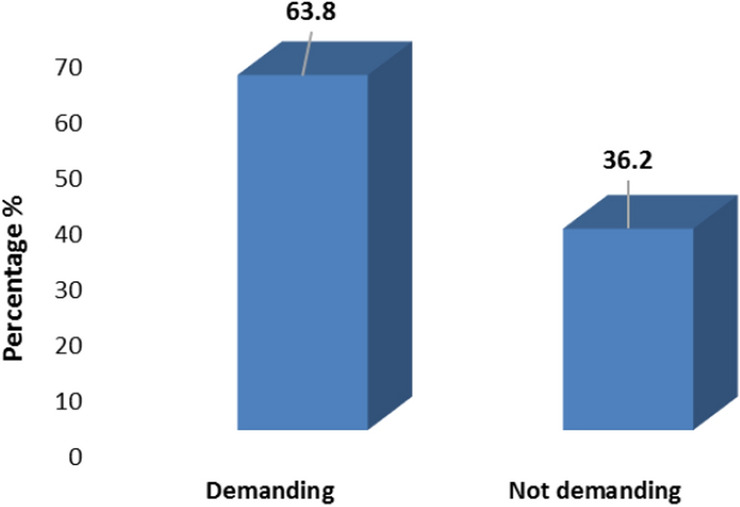
Percentage of demand and not demand for mammography at the initial bid

Based on Fig. 1, 63.84% (95% CI 58.68–68.70) of people demanded mammography at the initial bid.

Table 3 indicates the rate of demand and non-demand for mammography based on the offered prices. As expected, the demand rate is lower at higher prices and higher at lower prices.

**Table 3:** Rate of demand for mammography based on the offered prices

Demand	Bid		
	(1.17 US$)	(3.33 US$)	(9.67 US$)
NO	21.19%	30.17%	56.67%
Yes	78.81%	69.83%	43.33%

At the time of data collection, each US dollar was equivalent to 600,000 Iranian Rial

**Fig. 2:**
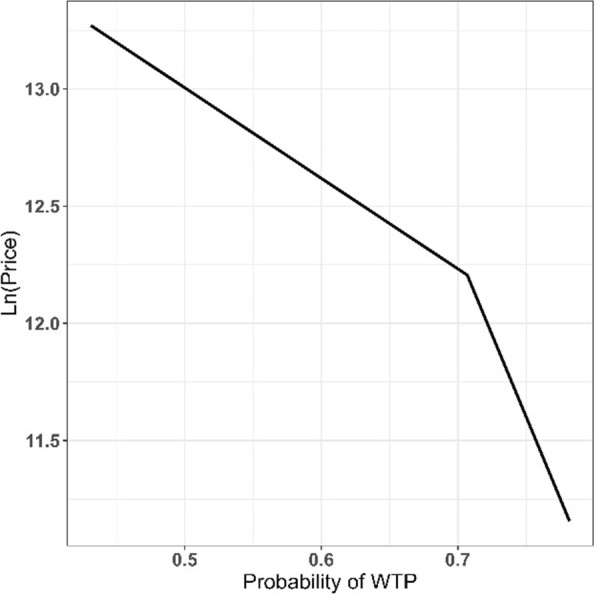
Mammography demand curve of participants

Figure 2 depicts participants’ mammography demand curve. The vertical axis determines the natural logarithm of the initial offer price based on Iranian US$, and the horizontal axis displays the probability of WTP the offered bid. As it is shown, demand is more elastic at higher prices than at lower prices.

Based on the results of Table 4, the price elasticity of demand for the test is estimated at − 0.19 (*P* < 0.01); with a 1% increase in the initial bid, the participant’s demand for mammography decreases by about 0.19%. Income elasticity of demand was calculated to be 0.24 (*P* < 0.01).

**Table 4:** The results of estimating price and income elasticity of mammography demand

Demand	dy/dx	Robust SE	*P* value	95% CI	
LnP	− 0.19	0.03	< 0.01	− 0.25	− 0.13
LnIncome	0.24	0.05	< 0.01	0.13	0.34
N = 349, Wald chi^2^(2) = 49.82, Prob > chi^2^ = 0.00, Pseudo R^2^ = 0.12					

### The effect of information framing on mammography demand

**Fig. 3:**
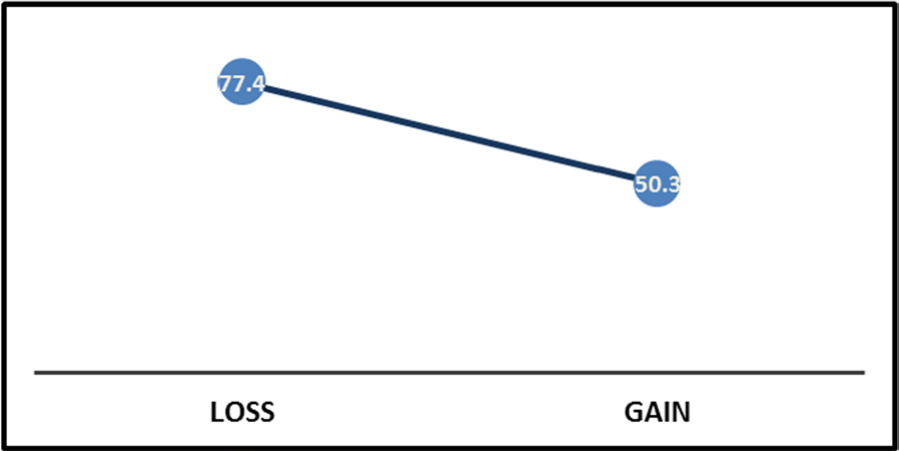
Participant mammography demand rate by frame type (%)

Figure 3 illustrates the mammography demand rate based on the type of information framing. In the loss frame, 77.40% of the participants, and in the gain frame, 5.30% of them demanded mammography.

In Table 5, the results of comparing the demand rate between two types of framing are reported based on the chi-square test. As it is shown, the higher demand rate in the loss frame compared to gain (38.7% vs. 25.1%) is statistically significant, and its effect size is estimated at 0.282 (*p* < 0.01).

**Table 5:** results of the comparison of the demand rate between two types of frame (chi-square test)

Statistics	Value	df	*p* value	Eta	Cohen’s D
Continuity correction	27.032	1	< 0.01	0.282	0.2

### Estimating the willingness to pay

Table 6 presents the estimated values of WTP for mammography across different models. In the first model, WTP was estimated using a basic demand model that considers only the bid price and income, yielding a WTP value of 8.23 USD. In the second model, WTP was calculated after adjusting for the framing effect. The estimates were further stratified by framing type, revealing that the WTP in the loss frame (13.09 USD) was significantly higher than in the gain frame (4.74 USD). Importantly, the confidence intervals for the WTP estimates in the loss and gain frames do not overlap, as indicated by López-Feldman [31]. This non-overlap confirms that the difference in WTP between the two framing models (study arms) is statistically significant at the 5% error level.

**Table 6:** The results of willingness to pay for mammography by model type

Model	WTP (US$)	SE	*P* value	95% CI	
Base model^*^	8.23	0.79	< 0.01	6.66	9.79
N = 354, Wald chi^2^(2) = 51.79, Prob > chi^2^ = 0.00, Pseudo R^2^ = 0.13					
Adjusted for framing effect	8.33	0.75	< 0.01	6.85	9.82
N = 354, Wald chi^2^(1) = 61.35, Prob > chi^2^ = 0.00, Pseudo R^2^ = 0.15					
Gain frame	4.74	0.91	< 0.01	2.96	6.52
N = 177, Wald chi^2^(1) = 15.43, Prob > chi^2^ = 0.00, Pseudo R^2^ = 0.06					
Loss frame	13.09	1.51	< 0.01	10.13	16.06
N = 177, Wald chi^2^(1) = 23.12, Prob > chi^2^ = 0.00, Pseudo R^2^ = 0.13					

At the time of data collection, each US dollar was equivalent to 600,000 Iranian Rial

*Basic demand function: The demand for mammography is considered a function of the offered price and income

## Discussion

This study aimed to understand the health message and information with which frame leads to the demand and perceived benefit of mammography screening. A semi-experimental study was conducted to answer this question.

### Mammography demand estimation

The findings revealed that about 63% of the participants demanded the mammography at the offered price. Available evidence shows varying rates for mammography demand. Wagner et al. [36]and Ghaderi et al. [37] have reported lower demand rates. While in Khaliq et al. [38], Bi et al. [39], Karimabadi et al. [40], Pokora et al. [41], and Zhang et al. [42] demand rate for mammography was higher than in our study. The difference in the rate of mammography demand between our research and other studies can be due to the differences in the type of screening, the study population, and other variables such as participants’ demographic and socioeconomic characteristics. We obtained a low price elasticity for mammography demand, and demand was more elastic at higher prices than at lower prices; changes in the offered price did not make much difference in the yes answer to the WTP question. Contrary to our findings, published evidence emphasizes the critical role of cost in mammography demand. A study of cancer screening in China concluded that most rural residents were only willing to pay a small fraction of the total cost of screening [43]. Tran et al. [44] disclosed that any out-of-pocket mammography reduces the probability of screening in the next 12–24 months by 3.0% points. Trivedi et al. [45] showed by difference-in-difference estimates that after removing cost sharing, the biennial mammogram rate for women increased by 6.5% points. Jena et al. [46] indicated that eliminating cost-sharing for mammography screening was associated with a slight decrease in screening rates among women. One reason for our inconsistent findings can be the difference in the study design. We elicited the participant’s demand for the bid price through the questions in which people may not give answers corresponding to the real situation. People’s actual demand between different randomized groups has been investigated in these studies. People’s responses in the simulated situation may not match their actual behavior. The demand for mammography was less elastic than the participants’ income. The income differences between the participants did not have much effect on their demand decision. This result can be due to these reasons. Maybe the offered price was too small compared to the participants’ purchasing power. Other covariates may have confounded the relationship between income and mammography demand. The participants may have unrealistically given positive answers to the WTP questions because of serving the researcher or because the conditions are not realistic. We could not find empirical evidence on the income elasticity of demand for mammography. Most studies have investigated the relationship between wealth and income with screening. Contrary to our finding (low-income elasticity of demand for mammography), most studies have shown that differences in income and wealth are critical in mammography uptake. For example, Zhang et al. [42] found that higher-income individuals were more likely to be willing to pay for a package of cancer screening services, including mammography. Pokora et al. [41] showed that mammography rates were higher among women with higher incomes. Nduka et al. [47] revealed that utilization of breast cancer screening is concentrated among wealthier women. The difference in the impact of income on mammography between our study and other studies can be due to these reasons. Instead of examining the demand for mammography, most studies have investigated the rate of uptake and utilization, and the initial price was not offered to the participants.

### The effect of information framing on demand and willingness to pay mammography

The mammography demand rate was statistically different between the two frames (loss and gain), so the rate of mammography demand in the loss frame is about 54% higher than in the gain frame. The value of WTP had a statistically significant difference between the two framings. Therefore, the value in the loss frame was estimated to be more than twice that of the gain frame.

Prospect theory [22] suggests that losses are more prominent than gains in human judgment. Our findings are consistent with Banks et al. [50], OKeefe & Jensen [25], Ferrer et al. [51], Shen & Mercerkolar [52], and Ainiwaer et al.[21]. In line with our findings, previous studies have shown greater effectiveness of loss-framed messages in promoting cancer screening behaviors such as breast self-examination [52], mammography [50], and colorectal cancer screening [51]. Appraisal of breast cancer screening interventions has shown that loss-framed messages are more effective than gain-framed messages [23, 43]. Banks et al. [50] revealed that within 12 months after the intervention, women who observed the loss message were more likely to have a mammogram than women who received the gain message. Abood et al. [53] showed that the chance of mammography is higher in women who received a loss framing message than in those who received a usual message. Considine et al. [54] concluded that loss frame telephone intervention provoked more screening than the gain frame. Finney & Lannotti disclosed that loss framing is superior to gain framing for inducing demand for mammography. For screening measures, loss messages elicited more positive perceptions of efficacy than gain messages [55]. For example, O’Keefe and Jensen’s [25] meta-analysis showed that loss-framed messages were more effective than gain-framed messages in promoting breast cancer screening behaviors but were not influential in encouraging other screening behaviors. However, another meta-analysis did not find a significant persuasive advantage of loss-framed messages in encouraging detection behaviors [23].

It has been argued that detection actions are more effectively encouraged by loss-framed messages than gain-framed messages [48, 49]. In fact, due to humans’ inherent tendency toward loss aversion, the late detection of cancer resulting from a lack of demand for screening can result in substantial losses, including worsened health outcomes and higher treatment costs. Screening plays a critical role in mitigating these losses by facilitating the early detection of cancer, thereby improving health conditions and reducing the overall burden of the disease. Several studies have investigated the effect of this framing on health behaviors. Gallagher & Updegraff [23] found in a meta-analysis that gain-framed messages encouraged prevention behaviors such as skin cancer prevention, smoking cessation, and physical activity more than loss-framed messages. Maltz et al. [24] concluded that there was no significant difference in the rate of medical checkup uptake between the gain and loss frames. The evidence obtained from studies is mixed, and the effectiveness of framing based on perspective theory in health is controversial [23, 25, 26].

### Factors that make a difference in the effectiveness of information frames

Various situational factors can affect the type of message frame effectiveness [56, 57]. Ainiwaer et al. [21] indicated that using loss-frame messages improves cancer detection behavior, and effectiveness weakens over time. Keysar et al. [58] showed that the framing effect disappears when describing information in a non-native language. It has also been concluded that as children age, the effects of framing in decision-making become stronger [59–61]. The perceived risk associated with a health action can influence the type of frame effectiveness. Rothman and Salovey [49] have argued that gain-framed messages may be more effective than loss-framed messages to promote health behaviors perceived as minimally risky. When individuals perceive health behaviors as high-risk, loss-framed messages often prove to be more effective. The function of the health behavior can influence the effectiveness of the framing type (gain or loss). For behaviors like mammography, which serve a detection function and are associated with a high degree of perceived risk, loss-oriented messages that induce loss aversion tend to have a stronger motivational impact. The higher the perceived risk of loss associated with a given prospect—such as the adverse consequences of not undergoing timely mammography—the greater the likelihood that individuals will demand mammography. According to the Health Belief Model, perceived risk is defined as the negative consequences of failing to perform health behaviors, such as mammography. Therefore, the more an individual perceives themselves to be at risk of loss due to inaction (e.g., not undergoing mammography), the more effective a loss-framed message is likely to be in promoting that behavior. It is important to note that participants in this study were randomly assigned to either the gain-framed or loss-framed message group. This randomization process ensured that variables like perceived risk, which could otherwise influence willingness to pay, were equally distributed across the two groups. Thus, the observed differences in willingness to pay between the two study arms can be attributed solely to the type of message format used (gain or loss). Regarding diagnostic behaviors such as mammography, people may have a more variable perceived susceptibility to their health status. In support of this argument, Apanovich et al.[68) showed that in promoting demand for HIV tests, loss-framed messages were more effective than benefit-framed messages only for people who were uncertain about the test result. For people who were confident that the test would not detect HIV, gain-framed messages were more effective in promoting testing than loss-framed messages. Similar results were also reported by Maheswaran & Meyerslevy [62] and Gallagher & Updegraff [23]. Evidence has confirmed the effect of framing in decision-making [55]. Health decisions can have lifelong well-being effects. Therefore, the framing effect can have various consequences on people’s well-being. People’s decision-making may be biased due to framing, which causes them to make suboptimal choices. For example, the inappropriateness of the frame in which health information and messages are presented to people may cause them not to demand cancer screening. In cancer, framing can shift the focus of older adults from short-term survival to long-term survival [63]. After the initial choice, a change in the description of the options can cause people to change their initial decision in favor of an alternative option [64].

### Comparison of methods for estimating willingness to pay

Several methods are available for eliciting WTP [65]. These methods include the open-ended question format, payment scales, closed-ended questions, and the double-bounded dichotomous bidding method. Each method has its own strengths and limitations. The open-ended question method, in which demanders directly state the maximum price they are willing to pay, is straightforward and simple to implement. However, it is often criticized for its lack of alignment with real-world market dynamics. In actual markets, individuals cannot acquire a product simply by stating a price, and responses in this format may be unrealistic or hypothetical. In contrast, the double-bounded dichotomous bidding method, also known as iterative bidding, is considered a more accurate representation of real market conditions. In this approach, respondents are first presented with an initial bid price. Based on their response (affirmative or negative), the bid is either increased or decreased, continuing iteratively until the respondent’s willingness to pay is determined. While this method is advantageous in its ability to closely mimic real-world scenarios, it is often criticized for being cognitively demanding for respondents, requiring more time and effort to complete, and potentially leading to respondent fatigue or confusion. For the purposes of our study, the closed-ended question method was selected. This method was chosen for its simplicity and its ease of understanding for participants. Unlike open-ended questions, the closed-ended approach presents respondents with a fixed price for the product and allows them to make a binary decision—either to accept or reject the price. This format not only reduces cognitive burden for participants but also aligns more closely with actual market behavior, where buyers typically decide whether to purchase a product at a given price.

## Limitations

We acknowledge that our study has some limitations. First, we examined the effect of framing on demand cross-sectionally and without time intervals. Therefore, we suggest that prospective studies address this limitation through longitudinal analysis. Secondly, we used the stated demand instead of examining the actual demand for mammography. We suggest that future studies investigate the effect of health information framing on the actual performance of mammography. Thirdly, our study was quasi-experimental, and we recommend that researchers address this issue through randomized controlled trial designs. Fourth, it is also likely that participants provide socially desirable answers to the willingness to pay question when surveyed face-to-face. Finally, the sample of participants in this study was drawn from a population survey conducted in the city of Qazvin. As a result, the findings of this study are specific to the population of Qazvin and cannot be generalized to other populations. The authors acknowledge this limitation and do not claim that the results are applicable beyond this context. Consequently, future research should explore this issue further by examining diverse populations in different geographic or cultural settings to enhance the generalizability of the findings. 

## Conclusion

Our findings revealed that the loss frame has a more persuasive effect than the gain frame; participants faced with the loss frame had more demand and WTP for mammography than the gain frame. Therefore, we suggest that health educators consider the type of health action persuasiveness before designing health messages. Educators also should use health messages with a loss frame to increase the demand for screening services such as mammography. 

## Supplementary Information

Supplementary material 1.

Supplementary material 2.

Supplementary material 3.

Supplementary material 4.

Supplementary material 5.

## Data Availability

The datasets used and/or analyzed during the current study available from the corresponding author on reasonable request. The entire dataset is in Farsi language. The Data can be available in English language for the readers and make available from the corresponding author on reasonable request.

## Footnotes

1 - To control underreporting of monthly income by individuals.

2 - Price elasticity is the percentage change in the probability of demand for a good relative to the percentage change in its price.

3 - Income elasticity is the percentage change in the probability of demand for a good as a percentage change in an individual's income.
