# Supplementary material for: The effect of promotional health message framing on the perceived benefit of mammography: evidence from estimation of willingness to pay
Source: J Health Popul Nutr. 2025 Jun 21;44:221. doi: 10.1186/s41043-025-00970-8 (PMC12182699; doi:10.1186/s41043-025-00970-8)
Supplement: Supplementary file 1 — Supplementary material 1. [file 41043_2025_970_MOESM1_ESM.pdf]

با سلام و احترام

پرسشنامه پیشروی جنابعالی به منظور جمع آوری اطلاعات مورد نیاز طرح تحقیقاتی "بررسی تقاضای ماموگرافی در بین زنان در معرض خطر شهر قزوین" تدوین شده است. این طرح توسط **دانشگاه علوم پزشکی قزوین** انجام می شود. حسن نظر و مشارکت دقیق شما برای نتایج این تحقیق بسیار ارزشمند است. قابل توجه است که مشارکت در این تحقیق کاملا **اختیاری** بوده و اطلاعات گردآوری شده **بدون نام** و **کاملا محرمانه** است. پیشاپیش از مشارکت شما بسیار سپاسگذاریم.

سن:	سطح تحصیلات:	قومیت:	وضعیت تاهل: ....
وضعیت اشتغال:<br>بیکار☐<br>شاغل☐	وضعیت بیمه درمانی پایه:<br>دارای بیمه☐<br>بدون بیمه☐<br>آیا بیمه مکمل دارید؟<br>بلی☐<br>خیر☐	مقدار درآمد ماهانه:<br>....................................	مقدار هزینه ماهانه (با در نظر گرفتن اجاره مسکن، خوراک، پوشاک، اقساط و غیره):<br>...

سوال 1)در حال حاضر وضعیت اقتصادی خود یا خانواده خود را در چه حدی ارزیابی می کنید؟

خیلی خوب☐ خوب☐ متوسط☐ ضعیف☐ خیلی ضعیف☐

سوال 2)از دوستان یا بستگان شما آیا کسی به سرطان پستان مبتلا بوده است؟

بلی☐ خیر☐ نمی دانم☐

سوال 3)چند درصد احتمال می دهید در آینده به سرطان پستان مبتلا شوید؟............

سوال 4)آیا در طی یکسال گذشته ماموگرافی انجام داده اید؟

بله☐ خیر☐ (بروید سوال 5)

سوال 5)آیا قصد دارید طی یکسال آینده ماموگرافی را انجام دهید؟

بله ☐ (بروید سوال 6) خیر☐

سوال 6)چقدر احتمال دارد که در یکسال آینده ماموگرافی را انجام دهید؟

خیلی کم☐ کم☐ متوسط☐ زیاد☐ خیلی زیاد☐

Appendix A1

<table>
<tr><td colspan="4">Greetings and regards
Your Excellency, the questionnaire has been developed to collect the information required for the research project "Investigating the demand for mammography among women at risk in Qazvin city". This project is being carried out by Qazvin University of Medical Sciences. Your kind opinion and detailed participation are very valuable for the results of this research. It is noteworthy that participation in this research is completely voluntary and the information collected is anonymous and completely confidential. We thank you very much in advance for your participation.</td></tr>
<tr><td>Age: ….</td><td>Literacy: ….</td><td>Ethnicity: ….</td><td>Marital status: …</td></tr>
<tr><td>Employment status:
Unemployed□
Employed□</td><td>Basic health insurance status:
Insured□
Uninsured□
Do you have supplementary insurance?
Yes□
No□</td><td>Monthly income: ….</td><td>Monthly expenses (including rent, food, clothing, installments, etc.):</td></tr>
<tr><td colspan="4">Question 1) How do you currently assess your or your family's economic situation?
Very good□ Good□ Average□ Poor□ Very poor□
Question 2) Has anyone among your friends or relatives been diagnosed with breast cancer?
Yes□ No□ Don't know□
Question 3) What is the probability that you will be diagnosed with breast cancer in the future?
Question 4) Have you had a mammogram in the past year?
Yes• No• (Go to Question 5)
Question 5) Do you plan to have a mammogram in the next year?
Yes• (Go to Question 6) No•
Question 6) How likely is it that you will have a mammogram in the next year?
Very low• Low• Average• High• Very high•</td></tr>
</table>

Appendix A2

Region	Parks name
1	Sabze meydan, Bostan-e- shahid motahhari
2	Banovan, Navvab-e- shomali, Mellat
3	Orcide, Lale, Pamchal
