# Supplementary material for: The effect of promotional health message framing on the perceived benefit of mammography: evidence from estimation of willingness to pay
Source: J Health Popul Nutr. 2025 Jun 21;44:221. doi: 10.1186/s41043-025-00970-8 (PMC12182699; doi:10.1186/s41043-025-00970-8)
Supplement: Supplementary file 2 — Supplementary material 2. [file 41043_2025_970_MOESM2_ESM.pdf]

**چارچوب منفعت**
**اگر به طور منظم و سر موقع ماموگرافی انجام دهید:**<br>❖ سرطان پستان **زودتر** و در مراحل **خفیف تر** و **قابل درمان تر** در شما پیدا می شود.<br>❖ **شانس زنده ماندن** شما **افزایش** می یابد.<br>❖ **انجام دادن به موقع ماموگرافی** می تواند **جان شما را نجات دهد.**<br>❖ نسبت به بانوانی که به موقع ماموگرافی انجام نمی دهند **25 تا 30 درصد بیشتر** **ایمن هستید**.<br>❖ **شانس زندگی در کنار خانواده تان** را **افزایش** می دهید.
**چارچوب زیان**
**اگر به طور منظم و سر موقع ماموگرافی انجام ندهید:**<br>❖ سرطان پستان **دیرتر** و در مراحل **شدیدتر** در شما پیدا می شود.<br>❖ **شانس مردن** شما بخاطر سرطان پستان **افزایش** می یابد.<br>❖ **انجام ندادن به موقع** ماموگرافی ممکن است **به قیمت جان شما** تمام شود.<br>❖ **25 تا 30 درصد بیشتر** از بانوانی که به موقع ماموگرافی انجام می دهند **در معرض خطر** هستید.<br>❖ ممکن است **زودتر** خانواده خود را **تنها بگذارید.**

Gain frame

If you have regular and timely mammograms:

 Breast cancer is found earlier and at a milder, more treatable stage.
 Your chances of survival increase.
 Having a mammogram on time can save your life.
 You are 25 to 30 percent safer than women who do not have a mammogram on time.
 You increase your chances of living with your family.

Loss frame

If you **don't get regular and timely** mammograms:

 Breast cancer will be found later and at a more advanced stage.
 Your chances of dying from breast cancer increase.
 Not getting a mammogram on time may cost you your life.
 You are at 25 to 30 percent more risk than women who get mammograms on time.
 You may leave your family alone sooner.
