# Supplementary material for: The effect of promotional health message framing on the perceived benefit of mammography: evidence from estimation of willingness to pay
Source: J Health Popul Nutr. 2025 Jun 21;44:221. doi: 10.1186/s41043-025-00970-8 (PMC12182699; doi:10.1186/s41043-025-00970-8)
Supplement: Supplementary file 3 — Supplementary material 3. [file 41043_2025_970_MOESM3_ESM.pdf]

فرض کنید به متخصص زنان و زایمان مراجعه کردید و او برای شما نوشته که باید ماموگرافی انجام دهید. شما مطلع شدید که هزینه انجام ماموگرافی .... است. آیا با این هزینه ماموگرافی را انجام می دهید؟

بلی انجام می دهم □

خیر انجام نمی دهم □

Suppose you visit an obstetrician and gynecologist and he writes to you that you need to have a mammogram. You are informed that the cost of a mammogram is .... Would you have the mammogram at this cost?

Yes, I will □

No, I will not □
