# Supplementary material for: The effect of promotional health message framing on the perceived benefit of mammography: evidence from estimation of willingness to pay
Source: J Health Popul Nutr. 2025 Jun 21;44:221. doi: 10.1186/s41043-025-00970-8 (PMC12182699; doi:10.1186/s41043-025-00970-8)
Supplement: Supplementary file 4 — Supplementary material 4. [file 41043_2025_970_MOESM4_ESM.pdf]

Qazvin University of Medical Sciences

Research Ethics Committees Certificate

<table>
<tr><td>Approval ID:</td><td>IR.QUMS.REC.1402.371</td><td rowspan="2">Approval Date:</td><td rowspan="2">2024-02-18</td></tr>
<tr><td>Evaluated by:</td><td>Research Ethics Committees of Qazvin University of Medical Sciences</td></tr>
<tr><td>Status:</td><td colspan="3">Approved</td></tr>
<tr><td>Approval Statement:</td><td colspan="3">The project was found to be in accordance to the ethical principles and the national norms and standards for conducting Medical Research in Iran.

Notice:
1. Although the proposal has been approved by the Biomedical Research Ethics Committee, meeting the professional and legal requirements is the sole responsibility of the PI and other project collaborators.
2. This certificate is reliant on the proposal/documents received by this committee on 2024-02-18. The committee must be notified by the PI as soon as the proposal/documents are modified.</td></tr>
<tr><td>Proposal Title:</td><td colspan="3">Investigating the impact of health information framing on the perceived value of mammography among females at risk in Qazvin city</td></tr>
<tr><td>Principal Investigator:</td><td colspan="3">Name: bahman ahadinezhad
Email: Bahmanahadi2009@gmail.com</td></tr>
</table>

Dr. Abdollah Didban
Committee Director
Qazvin University of Medical Sciences

Dr. Seyyed Mehdi Mirhashemi
Committee Secretary
Qazvin University of Medical Sciences
