# Supplementary material for: The effect of promotional health message framing on the perceived benefit of mammography: evidence from estimation of willingness to pay
Source: J Health Popul Nutr. 2025 Jun 21;44:221. doi: 10.1186/s41043-025-00970-8 (PMC12182699; doi:10.1186/s41043-025-00970-8)
Supplement: Supplementary file 5 — Supplementary material 5. [file 41043_2025_970_MOESM5_ESM.pdf]

Table E1: Comparing participant characteristics between the two gain and loss arms

variable		Value		P_value
		frame		
		Gain	Loss	
Age		$54.55±8.43^{a}$	$53.44±9.14^{a}$	0.23
Income (US$)		$19.60±12.56^{a}$	$20.82±12.34^{a}$	0.37
Perceived risk (%)		$7.64±20.67^{a}$	$3.90±14.67^{a}$	0.07
Employment status	Jobless (%)	79.10^{b}	69.50^{b}	0.05
	Employed (%)	20.90^{b}	30.50^{b}	
Health insurance	No (%)	5.10^{b}	6.20^{b}	0.82
	Yes (%)	94.90^{b}	93.80^{b}	
Perceived economic status		166.75^{c}	179.21^{c}	0.21
Literacy level		168.27^{c}	186.73^{c}	0.08
a: Mean ± SD, b: Frequency and c: Mean rank				

WTP estimation for basic model of demand

```
. probit wtp income bid, vce(robust)


Iteration 0:   log pseudolikelihood = -231.63032
Iteration 1:   log pseudolikelihood = -204.64742
Iteration 2:   log pseudolikelihood = -201.62083
Iteration 3:   log pseudolikelihood =  -201.5824
Iteration 4:   log pseudolikelihood =  -201.5824

Probit regression                    Number of obs =    354
                                     Wald chi2(2)  =  51.79
                                     Prob > chi2   = 0.0000
Log pseudolikelihood = -201.5824     Pseudo R2     = 0.1297

------------------------------------------------------------------------------
             |               Robust
         wtp | Coefficient  std. err.      z    P>|z|     [95% conf. interval]
-------------+----------------------------------------------------------------
      income |   5.44e-08   1.72e-08     3.17   0.002     2.08e-08    8.80e-08
         bid |  -2.09e-06   3.23e-07    -6.46   0.000    -2.72e-06   -1.45e-06
       _cons |   .3382535   .2205819     1.53   0.125    -.0940791     .770586
------------------------------------------------------------------------------
Note: 0 failures and 1 success completely determined.


. nlcom (WTP:- (_b[_cons]+income_m*_b[income])/_b[bid]),noheader

------------------------------------------------------------------------------
         wtp | Coefficient  Std. err.      z    P>|z|     [95% conf. interval]
-------------+----------------------------------------------------------------
         WTP |   493625.3   47828.45    10.32   0.000     399883.3    587367.4
------------------------------------------------------------------------------
```

WTP estimation for gain frame

```
probit wtp income bid, vce(robust)

Iteration 0:   log pseudolikelihood = -122.68423
Iteration 1:   log pseudolikelihood = -113.44828
Iteration 2:   log pseudolikelihood = -113.40263
Iteration 3:   log pseudolikelihood = -113.40262

Probit regression                          Number of obs =    177
                                   Wald chi2(2)  =  19.87
                                   Prob > chi2   = 0.0000
Log pseudolikelihood = -113.40262                Pseudo R2     = 0.0757

------------------------------------------------------------------------------
             |               Robust
         wtp | Coefficient  std. err.      z    P>|z|     [95% conf. interval]
-------------+----------------------------------------------------------------
      income |   2.31e-08   1.37e-08     1.69   0.091    -3.70e-09    4.98e-08
         bid |  -1.87e-06   4.52e-07    -4.13   0.000    -2.75e-06   -9.82e-07
       _cons |   .2638199   .2205551     1.20   0.232    -.1684602    .6960999
------------------------------------------------------------------------------



. nlcom (WTP:- (_b[_cons]+income_m*_b[income])/_b[bid]),noheader

------------------------------------------------------------------------------
         wtp | Coefficient  Std. err.      z    P>|z|     [95% conf. interval]
-------------+----------------------------------------------------------------
         WTP |   284925.9   52304.98     5.45   0.000     182410.1    387441.8
------------------------------------------------------------------------------
```

WTP estimation for loss frame

```
. . probit wtp income bid, vce(robust)

Iteration 0:   log pseudolikelihood = -94.585938
Iteration 1:   log pseudolikelihood = -74.625548
Iteration 2:   log pseudolikelihood = -62.484255
Iteration 3:   log pseudolikelihood = -61.720084
Iteration 4:   log pseudolikelihood = -61.718712
Iteration 5:   log pseudolikelihood = -61.718712

Probit regression                               Number of obs =    177
                                        Wald chi2(2)  =  21.39
                                        Prob > chi2   = 0.0000
Log pseudolikelihood = -61.718712                    Pseudo R2     = 0.3475

------------------------------------------------------------------------------
             |               Robust
         wtp | Coefficient  std. err.      z    P>|z|     [95% conf. interval]
-------------+----------------------------------------------------------------
      income |   1.83e-07   4.85e-08     3.78   0.000     8.81e-08    2.78e-07
         bid |  -2.97e-06   6.85e-07    -4.33   0.000    -4.31e-06   -1.63e-06
       _cons |  -.1966394    .390287    -0.50   0.614    -.9615879    .5683092
------------------------------------------------------------------------------
Note: 0 failures and 4 successes completely determined.
```

```
nlcom (WTP:- (_b[_cons]+income_m*_b[income])/_b[bid]),noheader

------------------------------------------------------------------------------
         wtp | Coefficient  Std. err.      z    P>|z|     [95% conf. interval]
-------------+----------------------------------------------------------------
         WTP |   785771.9    90799.9     8.65   0.000     607807.4    963736.4
------------------------------------------------------------------------------
```

```
Iteration 0:   log pseudolikelihood = -231.63032
Iteration 1:   log pseudolikelihood = -189.42798
Iteration 2:   log pseudolikelihood = -185.43251
Iteration 3:   log pseudolikelihood = -185.42036
Iteration 4:   log pseudolikelihood = -185.42036

Probit regression                          Number of obs =    354
                                           Wald chi2(3)  =  75.74
                                           Prob > chi2   = 0.0000
Log pseudolikelihood = -185.42036          Pseudo R2     = 0.1995

------------------------------------------------------------------------------
             |               Robust
         wtp | Coefficient  std. err.      z    P>|z|     [95% conf. interval[
-------------+----------------------------------------------------------------
       frame |   .8569081   .1504742     5.69   0.000      .561984    1.151832
         bid |  -2.29e-06   3.47e-07    -6.61   0.000    -2.97e-06   -1.61e-06
      income |   5.80e-08   1.71e-08     3.38   0.001     2.44e-08    9.16e-08
_       cons |  -.0191422   .2224902    -0.09   0.931     -.455215    .4169306
------------------------------------------------------------------------------
Note: 0 failures and 1 success completely determined.

------------------------------------------------------------------------------ .
         wtp | Coefficient  Std. err.      z    P>|z|     [95% conf. interval[
-------------+----------------------------------------------------------------
         WTP |   500073.6   45409.45    11.01   0.000     411072.7    589074.5
------------------------------------------------------------------------------

.
```
